# Therapeutic immersion: a single-subject study on virtual reality multisensory experiences for mitigating body disturbance in anorexia nervosa

**DOI:** 10.1007/s40519-025-01740-5

**Published:** 2025-03-26

**Authors:** Giulia Brizzi, Margherita Boltri, Rebecca Guglielmini, Gianluca Castelnuovo, Leonardo Mendolicchio, Giuseppe Riva

**Affiliations:** 1 Applied Technology for Neuro‐ Psychology Laboratory, IRCCS Istituto Auxologico Italiano, 20149 Milan, Italy; 2 U.O. dei Disturbi del Comportamento Alimentare, I.R.C.C.S. Istituto Auxologico Italiano, Ospedale San Giuseppe, 28824 Piancavallo, VCO Italy; 3 Experimental Laboratory for Metabolic Neurosciences Research, I.R.C.C.S. Istituto Auxologico Italiano, 28824 Piancavallo, VCO Italy; 4 Department of Psychology, Università Cattolica del Sacro Cuore, Largo Gemelli, 20121 Milan, Italy; 5 Clinical Psychology Research Lab, IRCCS Istituto Auxologico Italiano, Auxologico Piancavallo, Verbania, Italy; 6 Humane Technology Laboratory, Università Cattolica del Sacro Cuore, Largo Gemelli, 20121 Milan, Italy

**Keywords:** Virtual reality, Multisensory integration, Body, Anorexia nervosa, Body illusion, Mirror exposure, Egocentric, Allocentric

## Abstract

Anorexia Nervosa (AN) is a mental health condition marked by reduced food consumption, intense fear of weight gain, and distorted body experience. Virtual Reality (VR)-based techniques—i.e., Body-Swapping and Mirror Exposure techniques—offer promising avenues for addressing body disturbance in AN, allowing patients to embody virtual bodies varying in size and shape. We used a single-subject experimental design to explore the potential of virtual reality multisensory experiences for mitigating body disturbance in AN. We examine the case of a 24-year-old female hospitalized with restrictive Anorexia Nervosa who underwent a novel body-focused VR intervention alongside the hospital's standard care. The intervention included six bi-weekly sessions, wherein the patient was immersed in a VR environment that allowed her to embody a virtual body that matched a healthy weight range. Through interactions with a virtual mirror, the patient was encouraged to concentrate on the body's positive aspects and functional abilities, thinking about its significance in her past, current, and anticipated future activities (Functional Mirror Exposure). The patient reported reduced body dissatisfaction, negative beliefs, and body misperception. These changes endorse using immersive, multisensory, and embodied experiences to alter body consciousness and body–self relationship. Results preliminary support the transformative potential of VR in improving traditional approaches to assess and alter body disturbance.

*Level of evidence* Level IV.

**Supplementary Information:**

The online version contains supplementary material available at 10.1007/s40519-025-01740-5.

## Introduction

Anorexia nervosa (AN) is an Eating Disorder (ED) characterized by reduced food intake, extremely low body weight, fear of gaining weight, and Body Image Disturbance (BID). BID reflects an altered perceptual, cognitive, and affective body experience [1]. Notably, BID plays a critical role in AN development, maintenance, and relapse [1]. Because of the limited effectiveness of available AN treatment, previous authors pointed out the need to include BID-focused protocols in AN interventions (e.g., Cognitive Dissonance Mirror Exposure; CD–ME [2]). In this regard, Virtual Reality (VR) has been increasingly used to allow patients to enter (embody) a different body compared to their real one. Such procedures include Body Swapping (BS; [3]) and VR–Mirror Exposure (VR–ME; [4]). Keizer et al. [5] and Serino et al. [3] observed that swapping into a normal-weight body reduced body size overestimation in AN patients, suggesting that embodiment in an artificial body with different dimensions from an egocentric frame altered body perception congruently with the artificial body [3, 5]. Porras-Garcia et al. [4] used VR–ME to gradually expose patients affected by AN to a virtual normal-weight body from an allocentric spatial frame (i.e., mirror-like perspective), observing a reduction in patients’ fear of gaining weight and body-related anxiety after this exposure.

Alleva et al. [6] suggested a new way to address BID focusing on body functionality (i.e., the body’s sensory, creative, and social abilities). Following this suggestion, Warren and Murray [7] proposed an ME variant called Functionality-Focused Mirror Exposure (FME). Here, individuals are asked to reflect on what their bodies allow them to do in everyday life while looking at themselves in the mirror.

However, no study has integrated this concept into VR approaches. By merging functionality-focused principles with VR-based interventions, this study aims to offer an innovative method to address BID, leveraging the strengths of different techniques.

As suggested by [8], the single-case methodology represents a first step in developing evidence-based practices and can be used to investigate a treatment's preliminary effectiveness. Here, we present a case study aimed at investigating the feasibility, acceptability, and preliminary efficacy of a new BID intervention in a patient affected by AN. We call it “VR-Functional Mirror Exposure” (VR-FME), and it was designed to include a combination of CD–ME and FME in VR. This design might allow the evaluation of the intervention’s feasibility and acceptability both from the patient’s and clinician’s point of view, promoting valuable insights to inform subsequent Randomized Control Trials.

### Case formulation

The patient was a 24-year-old woman. She started restricting her food intake in 2014, reporting that her family encouraged her to control her body weight and diet. Food restriction led to significant weight loss and a first hospitalization in 2015 with a diagnosis of restrictive AN. In 2016, she entered a center specializing in ED. In the following years, the patient reported a fair psycho-physical well-being. Restricted eating, purging episodes, and motor hyperactivity reemerged in 2023, coinciding with the transfer to another city for studying. She sought admission in 2024 to the IRCCS (Istituto di Ricovero e Cura a Carattere Scientifico) Istituto Auxologico Italiano, with a BMI = 15.7 and a diagnosis of Restrictive AN. The hospital program includes a 4-week stay. During the hospitalization, a series of activities are offered (see Table S in Online Resources for the description of standard-intervention activities). The patient was introduced to an innovative protocol using VR experiences to address BID alongside the standard approach.

## Methods

### Procedure

**Fig. 1:**
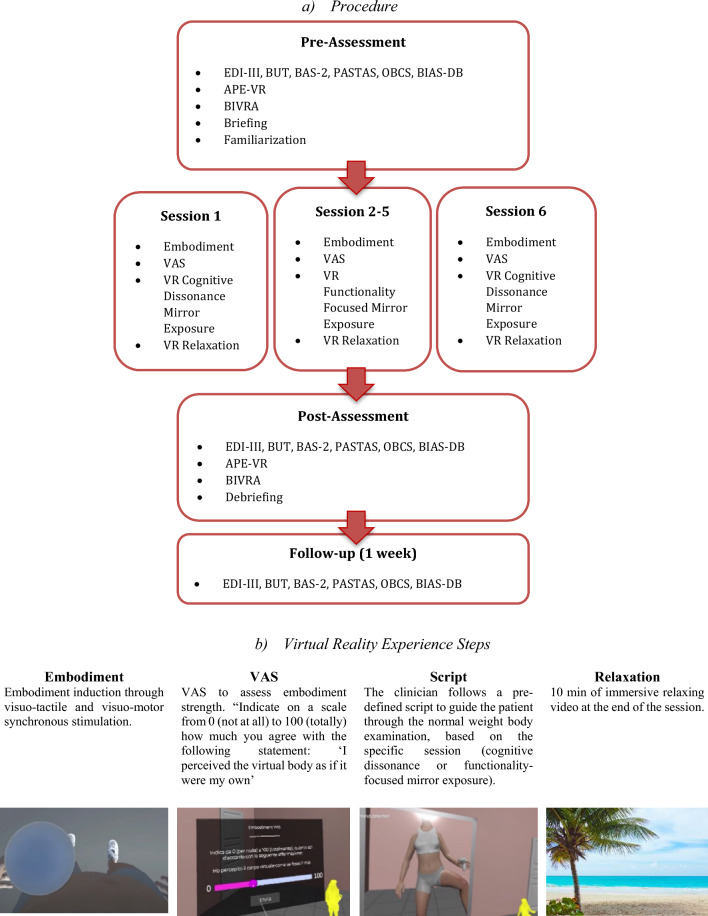
Procedure and Virtual Experience. **a** Represents the study procedure. Eating Disorder Inventory–3 (EDI-III), the Body Uneasiness Test (BUT), the Body Appreciation Scale-2 (BAS-2), the Objectified Body Consciousness Scale (OBCS), the Figure Rating Scale (BIAS–BD), the Aperture Task in Virtual Reality (APE-VR) and the Figure Rating Scale in Virtual Reality (BIVRA); Visual Analogue Scale (VAS), Virtual Reality (VR). Because of technical constraints, VR tasks were not proposed in the follow-up, and the BMI was not asked of the participant, following clinicians' suggestion. Online Resource contains a full description of the measures used, including psychometric information and details of scoring; **b** shows the VR experience core steps. The body model was developed using MakeHuman Software based on [1] and resembles a normal-weight body (BMI = 18.5)

The protocol consisted of 2 weekly sessions for a total of 6 meetings. A pre-assessment before the first session, a post-assessment after the last session, and a follow-up 1 week after the end of the intervention were conducted. In the pre-assessment, ED symptomatology and body disturbance were assessed. The patient was also asked about her expectations and attitudes toward VR. VR sessions started with the induction of a full body illusion (i.e., visuomotor and visuotactile stimulation for 90 s). Then, based on the session, CD–ME or FME exercises were proposed in VR. The same body model (BMI = 18.5) was presented throughout the sessions. Finally, the patient was immersed in a relaxing virtual scenario. In the post-assessment, pre-assessment measures were administered. Finally, thoughts about the experience were collected. In the follow-up*,* questionnaires were e-mailed to the patient (Fig. 1). An in-depth explanation of the procedure—including a session-by-session description and the script used in the different sessions—is available in Online Resources.

### Measures

The Eating Disorder Inventory-III (EDI-III) and Body Mass Index (BMI) were used to assess symptomatology. For the body–self relationship, the Body Uneasiness Test (BUT), Body Appreciation Scale-2 (BAS-2), Physical Appearance State and Trait Anxiety Scale (PASTAS), and Objectified Body Consciousness Scale (OBCS) were included. Body perception was evaluated using the Figure Rating Scale (BIAS–BD), the Body Image Virtual Reality Assessment (BIVRA), and Affordance Estimation Procedures in Virtual Reality (APE–VR). Finally, a Visual Analog Scale (VAS) assessed embodiment during VR sessions. See Online resources for additional information about assessment measures and scoring.

### Virtual reality intervention

*Cognitive dissonance mirror exposure (CD–ME)* was proposed in the first and last sessions. The exposure started with the patient standing in front of the virtual mirror. She was encouraged to describe out loud different body parts of the virtual body using positive or neutral words (e.g., the body's physical, emotional, and social qualities). For an individual with high body distress, complimenting oneself in the mirror creates cognitive dissonance. Notably, VR allows the application of this technique to a normal-weight body, unlike real-world procedures. During the last 5 min of the session, the patient was immersed in a relaxing natural VR environment. The session lasted around 60 min.

*Functional mirror exposure procedure (VR–FME)* was used in the central sessions. VR–FME was based on [6, 7], adapting the script to focus on critical body areas (i.e., the facial scan was removed to give more space to body areas, such as the hips and abdomen). The script encouraged the patient to verbalize some activities/experiences her body allowed her to do in the past and present and will allow her to do in the future. Outloud verbalization partially ensured she was paying attention to the task. She was also instructed not to avoid uncomfortable feelings or thoughts but to refocus gently on the instructions. The underlying idea was to prompt a different way to look at the body away from physical appearance, and create a connection between a healthy body and positive memories. The last 5 min of the session were dedicated to relaxation. Each session lasted approximately 60 min.

Online Resources provide detailed information about the VR procedures and the entire protocol structure, techniques, and/or tools.

## Results

### Quantitative data

EDI-III scores suggested a reduction across various eating disorder symptoms and related psychological factors. However, some subscales remained unchanged or showed minimal variation (Table 1). In general, the intervention seemed to influence more psychological features related to the disorder rather than ED-specific symptoms.

**Table 1:** EDI-III subscales scores in pre-, post-, and follow-up assessment points

Eating Disorder Inventory–III			
	*Pre*	*Post*	*Follow-up*
Drive for Thinness	28	28	28
Bulimia	2	1	3
Body Dissatisfaction	40	40	40
Low Self Esteem	23	28	24
Personal Alienation	20	10	14
Interpersonal Insecurity	5	1	0
Interpersonal Alienation	6	7	8
Interoceptive Deficits	10	4	4
Emotional Dysregulation	7	3	4
Perfectionism	20	20	20
Asceticism	17	16	12
Maturity Fears	32	30	32
Inadequacy Composite	43	28	38
Interpersonal Problems Composite	11	8	8
Affective Problems Composite	17	7	8
Overcontrol Composite	37	36	32
General Psychological Maladjustment Composite	140	109	118

In the pre-assessment, the patient’s BMI was 15.7. In the first session, it was 15.3, whereas after the intervention, BMI was 15.8.

Questionnaires assessing the body–self relationship showed improvements after the intervention (Table 2). The patient showed an overall reduction in body uneasiness, whereas fear of gaining weight and body image concerns did not. The number of areas causing distress decreased, although the distress intensity increased. In addition, the patient showed a higher body appreciation and reduced physical anxiety and self-objectification after the intervention.

**Table 2:** Body self-relationship questionnaires

	*Pre*	*Post*	*Follow-up*
BUT			
Global Severity Index	4.5	4.26	4.00
Weight Phobia	5.00	5.00	5.00
Body Image Concerns	4.44	4.44	4.44
Avoidance Behaviors	4.17	3.33	1.83
Image Control	4.17	4.17	4.00
Depersonalization	4.60	4.00	4.17
Positive Total Symptoms	18.00	15.00	16.00
Positive Symptom Distress Index	4.17	5.00	4.81
TOT A (whole body)	26.88	25.20	23.44
TOT B (specific body areas)	22.17	20.00	20.81
BAS-2			
	13	15	14
PASTAS			
	40	32	36
OBCS			
	128	132	126

Finally, the patient showed greater accuracy in the “real” body identification task in the post-assessment as compared to the pre-assessment. Notably, while no significant differences were found in the BIAS–BD, changes in ideal body shape were observed in BIVRA. It followed an overall improvement in body satisfaction. APE–VR indicated changes in the sensorimotor representation of the body.

Table 2*.* **a** Scores in the questionnaires assessing body–self relationship in the pre-, post-, and follow-up assessment points. Scores in the Body Uneasiness Test (BUT) were divided for subscales on the left side, and scores in the Body Appreciation Scale (BAS-2), Physical Appearance State and Trait Anxiety Scale (PASTAS), and Objectified Body Consciousness Scale (OBCS) were on the right side. Table 3 Scores in BIAS–BD, BIVRA, and APE_VR tasks in the pre-, post-, and follow-up assessment points. The VR task was reported as the mean value of responses to each trial. In the pre-assessment, BIVRA real selection corresponds to an avatar with a BMI = 19.5, whereas in the post-assessment, the real selection corresponds to an avatar with a BMI = 18.5. Ideal body selection in BIVRA showed that the ideal body shape in the pre-assessment corresponds to an avatar with a BMI of 18.5 and an avatar with a BMI of 19.5 in the post-assessment. More detailed information about the measures and scoring is available in Online Resources.

**Table 3:** Body perception and internalized self-concept

BIAS–BD				
Real	140	105	100	
Ideal	60	60	60	
Dissatisfaction	80	45	40	
BIVRA				
Real	2.00	1.20		
Ideal	1.50	1.00		
Dissatisfaction	1.20	0.20		
APE–VR				
	1.65	1.22		

Embodiment strength ranged from 100 (max) to 88.43 (min) across the sessions, suggesting high embodiment.

### Free feedback

#### Cognitive dissonance

In Session 1, the participant reacted with dissatisfaction with the virtual body's appearance, focusing on perceived flaws. She noted a positive aspect in the virtual figure's height, but overall, the negative focus dominated. By Session 6, the participant began to acknowledge the virtual body's positive aspects, such as a normal neck, a flat stomach, and strong legs and arms, viewing them as beneficial for swimming and walking. This marked a transition from focusing solely on aesthetics to appreciating the body's capabilities.

#### Virtual reality expectations and user experience

Before the intervention, the participant, concerned about body distortion, hoped it would help address this issue. Although new to VR, she was eager for the experience. After the sessions, she found the experience enjoyable and felt comfortable with the technology and experimenters. However, she was unsure of its impact on body image. She had expected more noticeable changes and suggested the intervention would be more impactful if the virtual body matched her current body. Despite this, she expressed a willingness to use VR in the future.

## Discussion

The current case study supports using VR–FME—a combination of CD–ME and FME in VR—to address BID in AN. Results revealed positive changes in altered body–self relationship, internalized self-concept, and improvements in psychological functioning.

### Anorexia nervosa symptomatology

While EDI-III psychological functional subscales showed improvements, ED risk scales remained stable—consistent with minimal BMI variation—differing from previous research [4]. However, the latter considered patients with lower symptom severity than this study. Therefore, understanding for whom and when the VR–FME might be more useful is needed. In addition, differences might be linked to the fact that we followed [5] and proposed a standard body instead of increasing the body BMI across sessions (i.e., starting from the patient’s BMI to arrive at a normal-weight body in the last session [4]). Furthermore, here we focused on body functionality [9]: we did not directly target weight-related anxiety or body satisfaction [4]. VR–FME is intended to achieve two goals: correcting the distorted body representation and offering a new way to relate to the body, suggesting this might impact different body–self components. Indeed, the VR experience can produce a sensory “surprise” and change the internal body model [1] and can restore a functional body–self relationship by linking a normal weight body to positive autobiographical memories [10].

### Body–self relationship

The patient showed improvements in BUT, differently from [10]. However, because the number of dissatisfying areas decreased but the total stress score did not, it could be that the intervention impacted medium/low critical body area judgments, while most critical areas remained distressing. It could also be that the level of uneasiness is accentuated when measured immediately after an emotionally demanding procedure such as body exposure. The decrease between pre-assessment and follow-up confirms this hypothesis. Another possibility is that to affect most critical areas, prolonged exposures are needed. Following the personalized medicine approach, an initial assessment could lead to tailored exposure time and script, which could be adjusted based on the patient's responses. Notably, we observed a reduction in self-objectification in the follow-up. While a rise in post-assessment was observed, this may reflect the experience's emotional impact. This aligns with [6], who suggested that changes in body perception can be slow but lasting. Like [3, 10], we observed that embodying and viewing a normal-weight body through mirror exposure reduced body-related anxiety. This reduction likely stems both from the exposure itself and the script’s focus on encouraging a more compassionate, positive view of the body. These findings support the idea that a body-functionality approach can improve the body–self relationship, expanding the body concept. Our results also differ from previous studies using writing tasks [7]: verbalizing may act as a catalyst for a functional-oriented body perception.

### Internalized self-concept

A reduction in body dissatisfaction was observed, as measured through avatar or silhouette selection and the aperture task. In line with [3, 5], the participant reduced body size overestimation after the intervention, demonstrating improved recognition of a body resembling her actual dimensions. This could be because embodying a different body might disrupt the brain’s internal model, aligning with predictive coding theory [1]. Notably, no change was noted in the ideal body estimation through silhouette selection, possibly due to response bias from presentation order. Indeed, the BIVRA variation underscores the limitations of traditional paper-and-pencil tasks.

### Free feedback

In the first session, the patient failed to report positive aspects of the virtual body. In the last session, she automatically focused on body functionality. This suggests that the work has promoted a different way of approaching the body. This further supports VR's ability to drive transformative experiences. Notably, overall feedback about the experience was positive. Starting from the patient’s feedback, future research might consider proposing body functionality exercises while changing the BMI of the virtual body at each session (i.e., starting from the current patients’ BMI to arrive at a normal weight body in the last session).

## Strengths and limits

This study presents some limitations. First, we cannot strongly conclude that the changes were due to VR exposure per se. A larger sample size and RCTs are needed to better understand the specific VR effects. Second, we considered only one follow-up assessment, but future research should consider different assessment points to detect changes over a longer period. Third, the BMI of the virtual body did not match the BMI of the patient, and we did not use a personalized body model, based on [12].

## What is already known on this subject?

VR body exposure showed promise in treating BID by allowing individuals to embody a different body from their own. However, while VR is a powerful tool, it is not inherently a treatment: specific tasks during exposure are necessary to enhance its potential therapeutic power.

## What does this study add?

This study offers preliminary evidence supporting the integration of VR–FME for BID in AN. Additional research is needed to better understand the clinical changes induced by this protocol on a larger sample size and in RCT.

## Supplementary Information

Below is the link to the electronic supplementary material.

Supplementary file 1.

## Data Availability

Data will be provided under request.
