# Supplementary material for: Therapeutic immersion: a single-subject study on virtual reality multisensory experiences for mitigating body disturbance in anorexia nervosa
Source: Eat Weight Disord. 2025 Mar 26;30(1):32. doi: 10.1007/s40519-025-01740-5 (PMC11946939; doi:10.1007/s40519-025-01740-5)
Supplement: Supplementary file 1 [file 40519_2025_1740_MOESM1_ESM.docx]

**Online Resources.**

**2. Methods**

**2.1 Protocol**

| 6 sessions + 1 pre-assessment, 1 post-assessment and 1 follow-up (after 1 week)  Twice per week, 60 minutes for each session | | |
| --- | --- | --- |
| Pre-Assessment measures*/ **** | Technology familiarization and procedure presentation. | - Questionnaires administration - Presentation of the procedure/ technology |
| Session 1 | [Stice and Presnell (2007)](https://www.sciencedirect.com/science/article/pii/S1740144511000441?casa_token=SPoEgcDtUX0AAAAA:3pQw9CPKvVB0MIpYqyTB01va_ANMDs1mo9fu9wIe7bqSBZZnuQWmS7ZynfSEjs4iUZ2BphE#bib0180) incorporated cognitive dissonance in mirror exposure sessions in an eating disorders prevention program. In their CD ME, participants described positive aspects of their physical, emotional, intellectual, and social qualities while looking at their image in a mirror. For an individual with body dissatisfaction, the act of complementing his or herself while looking in the mirror should induce cognitive dissonance. | - Embodiment procedure from first-person perspective (visuo-tactile stimulation for 90 seconds; Serino et al., 2016) – the body model has a BMI equal to 18.5 - Embodiment measures through a VAS (Porras-Garcia et al., 2021) - Cognitive Dissonance Mirror Exposure (CD ME; Luethcke et al., 2011): The body exposure task began by asking the patients to describe the body parts of their virtual body (starting from the head, shoulders, arms, hands, chest, stomach, waist, hips, thighs, lower legs, and then the feet). The clinician invited to participants describe positive aspects of their physical, emotional, intellectual, and social qualities while looking in the mirror. - Relaxation: the last 5 minutes of the session were dedicated to helping the patients reduce their anxiety or any other sort of discomfort that they might experience during the body exposure task. The patients were immersed in relaxing VR environments (i.e., waterfalls, forests, or beaches). |
| Session 2 | The Functionality-Focused Mirror Exposure Procedure (FME) procedure is based on the empirically supported body-functionality-focused body image intervention Expand Your Horizon (Alleva et al., 2015), which involves writing prompts about body functionality. Additionally, the current FME procedure expanded on the FME script described in Brooks and Walker (2017) to increase the FME’s duration and was based on an empirically supported mindfulness-based ME script.  Similar to other ME procedures, the FME leads participants to describe body parts, focusing on specific body parts and what they enable the individual to do and experience, with prompts that include physical and creative skills, physical, mental, and emotional capacities, interactions with nature (e.g., viewing beautiful scenery), and interpersonal relationships (e.g., speaking with a friend, hugging a family member or a pet). Patients were instructed to focus on facets of functionality for which they were grateful. They were also instructed not to avoid uncomfortable feelings or thoughts, but to gently refocus on the FME instructions. | - Embodiment procedure from first-person perspective (visuo-tactile) - Embodiment measures through a VAS - Functionality-Focused Mirror Exposure Procedure (FME; Walker & Murray, 2022) – below the script of the experience** - Relaxation: the last 5 minutes of the session were dedicated to helping the patients reduce their anxiety or any other sort of discomfort that they might experience during the body exposure task. The patients were immersed in relaxing VR environments (i.e., waterfalls, forests, or beaches). |
| Session 3 | This session continues and repeats the work started in the previous one. | - Embodiment procedure from first-person perspective (visuo-tactile) - Embodiment measures through a VAS - Functionality-Focused Mirror Exposure Procedure (FME) – 13 minutes ** - Relaxation: the last 5 minutes of the session were dedicated to helping the patients reduce their anxiety or any other sort of discomfort that they might experience during the body exposure task. The patients were immersed in relaxing VR environments (i.e., waterfalls, forests, or beaches). |
| Session 4 | This session continues and repeats the work started in the previous one. | - Embodiment procedure from first-person perspective (visuo-tactile stimulation for 90 seconds) - Embodiment measures through a VAS - Functionality-Focused Mirror Exposure Procedure (FME) – 13 minutes ** - Relaxation: the last 5 minutes of the session were dedicated to helping the patients reduce their anxiety or any other sort of discomfort that they might experience during the body exposure task. The patients were immersed in relaxing VR environments (i.e., waterfalls, forests, or beaches). |
| Session 5 | This session continues and repeats the work started in the previous one. | - Embodiment procedure from first-person perspective (visuo-tactile) - Embodiment measures through a VAS - Functionality-Focused Mirror Exposure Procedure (FME) – 13 minutes ** - Relaxation: the last 5 minutes of the session were dedicated to helping the patients reduce their anxiety or any other sort of discomfort that they might experience during the body exposure task. The patients were immersed in relaxing VR environments (i.e., waterfalls, forests, or beaches). |
| Session 6 | This session is equal to the first one to reinforce the work done in the previous meetings. | - Embodiment procedure from first- person perspective (visuo-tactiles; Serino et al., 2016) - Embodiment measures through a VAS (Porras-Garcia et al., 2021) - Cognitive Dissonance Mirror Exposure (CD ME; Luethcke et al., 2011): The body exposure task began by asking the patients to describe the body parts of their virtual body (starting from the head, shoulders, arms, hands, chest, stomach, waist, hips, thighs, lower legs, and then the feet). The clinician invited to participants describe positive aspects of their physical, emotional, intellectual, and social qualities while looking in the mirror. - Relaxation: the last 5 minutes of the session were dedicated to helping the patients reduce their anxiety or any other sort of discomfort that they might experience during the body exposure task. The patients were immersed in relaxing VR environments (i.e., waterfalls, forests, or beaches). |
| Post-Assessment measures (1^st^ follow up)* / **** |  | - Questionnaires administration - Debriefing |
| Post-Assessment measures (2^nd^ follow up)* |  | - Questionnaires administration |

**2.2 *Pre-Assessment, Post-Assessment, and Three Months Follow-Up Measures**

**Anorexia Nervosa Symptomatology**

*Eating Disorder Inventory -III (EDI-III;* Clausen et al., 2011*) –* the EDI-III is a self-report measure to assess psychological characteristics or features that have been demonstrated to be clinically significant in patients affected by Eating Disorders. It consists of 91 items organized into 12 primary scales: three eating disorder-specific scales (Drive for Thinness—DT; Bulimia—B; Body Dissatisfaction—BD) and nine general psychological scales (Low Self-Esteem—LSE; Personal Alienation—PA; Interpersonal Insecurity—II; Interpersonal Alienation—IA; Interoceptive Deficits—ID; Emotional Dysregulation—ED; Perfectionism—P; Asceticism—A; Maturity Fears—MF) that are highly relevant to, but not specific to, Eating Disorders. Each item on the EDI-III is rated on a 6-point response scale ranging from Always (1) to Never (6), with higher scores indicating more severe symptoms or psychological distress. The reliability coefficients of the scales range from 0.83 and 0.90, and test–retest reliability coefficients for the various composite scales are between 0.84 and 0.87. The Italian version of EDI-3 has demonstrated very good test–retest reliability, cross-informant agreement, and good discriminating validity (Cinelli et al., 2020).

*Body Mass Index (BMI) -* The change in body weight was evaluated each week by weighing the patient and calculating her BMI.

**Body -Self Relationship**

*The Body Uneasiness Test* (BUT; Cuzzolaro et al., 2006) – The BUT is a self-report questionnaire to measure body-related uneasiness. It consists of two main factors. The BUT-A consists of four subscales and a global severity index (GSI): Weight Phobia (WP—fear of being or becoming fat), Body Image Concerns (BIC—worries related to physical appearance), Avoidance (A—body image-related avoidance behavior), Compulsive Self-Monitoring (CSM—compulsive checking of physical appearance), and Depersonalization (D—detachment and estrangement feelings toward the body). Here respondents have to rate how much the 34 sentences represent them ranging from 0 (Never) to 5 (Always). BUT-B instead looks at specific worries about body parts or functions and consists of two subscales: the Positive Symptom Total (PST – the number of body areas that cause distress and discomfort) and Positive Symptom Distress Index (PSDI – the average rating of those items constituting PST). Here respondents have to rate how much they hate 37 different body areas from 0 (Never) to 5 (Always). Then, whereas BUT-A focuses on the whole body, BUT-B assesses uneasiness related to specific body areas. Higher scores in BUT-A and BUT-B index higher body-related concerns and distress. The Italian version of the instrument shows good reliability coefficients and a factorial structure congruent with the operative definition of the construct (Cinelli et al., 2020).

*Body Appreciation Scale-2* (BAS-2; Tylka & Wood-Barcalow, 2015) – The BAS-2 is a self-report measure of body appreciation, acceptance, favorable opinions, and respect. It consists of 10 items to which respondents respond by rating from 1 (Never) to 5 (Always) the extent to which the statements describe their body-self relationship. Higher scores indicate a greater appreciation of one's own body. The measure has been validated in Italian and shows good psychometric properties (Casale et al., 2021).

*Physical Appearance State and Trait Anxiety Scale – PASTAS* (Thompson, 1999) - The complete version of PASTAS comprises two self-report scales measuring weight-related and non-weight-related anxiety. In this study, the Trait-Weight Scale (W) was used in line with previous research (Porras-Garcia et al., 2020). It requires respondents to the level of anxiety related to different 16 body features and areas from 0 (Not at All) to 4 (Exceptionally So). Higher scores correspond to higher body appearance anxiety. In previous research, the scale was used in Italian samples (e.g., Malighetti et al., 2020).

*Objectified Body Consciousness Scale* (OBCS; McKinley & Hyde, 1996) – The OBCS is a self-report questionnaire assessing Objectified Body Consciousness. It is a 24-item questionnaire on a 7-point Likert scale (from 1 = strongly disagree to 7 = strongly agree) to evaluate the three main components of OBC: body surveillance, body shame, and control beliefs. Examples of items are “I rarely think about how I look”, “I would be ashamed for people to know what I really weigh”, and “I can weigh what I'm supposed to when I try hard enough”. Greater scores index a higher objectification level. We used this questionnaire since it has been validated in the Italian language, showing good psychometric properties and the same three-factor structures as the original English version (Dakanalis et al., 2017).

**Body Perception**

*Figure Rating Scale* (BIAS-BD; Garner et al., 2008) - The BIAS-BD is a depictive body-image assessment tool comprising 17 gender-specific contour-line drawings based on known anthropometric bodily dimensions. The drawings' body weights range from 60% below the established average to 140% over it. A 5% shift in body weight was represented by differences in figural drawings. Respondents are asked to select the figure that matches their current and ideal body shape and size.

*Body Image Virtual Reality Assessment* (BIVRA; Brizzi et al., 2024) – BIVRA is an immersive virtual reality body image rating scale consisting of 7 body stimuli ranging from underweight (1) to overweight (7). Respondents are at the center of a virtual room, and they are surrounded by virtual bodies (*Figure 2*). Here, similarly to BIAS-BD, they must select the stimulus that best matches their current and ideal body shape. Unlike BIAS-BD, the system presents the ideal/real choice task five times, manipulating the aesthetic features of the body models (i.e., eye and hair color) while holding body parameters constant. This allows to assess body percentual accuracy (i.e., the ability to select the virtual body with a BMI close to the real one) and body dissatisfaction (i.e., the difference between the ideal and real virtual body) as standard figure rating scales while avoiding explicit measures biases. BIVRA’s ability to assess body perception and body satisfaction has been demonstrated in previous research (Brizzi & Romano, 2022).

*Affordance Estimation Procedures in Virtual Reality* (APE-VR) – The Aperture Task (Irvine et al., 2019) is a metric task designed to assess body schema, namely the sensorimotor representation of one's body that is used to plan or guide actions. It requires participants to adjust the aperture of a door-like aperture to determine the minimal aperture they need to pass through without rotating their shoulders or hips. The APE-VR was proposed in a virtual environment where respondents had to adjust the opening of a virtual door four times, starting with the door fully closed or open. The virtual version has been previously observed to correlate with real door aperture estimation (Brizzi & Romano, 2022; *Figure 2*).

| BIVRA | APE_VR |
| --- | --- |
| Participants are immersed in a virtual room where they are surrounded by 7 different body models varying in BMI. The task requires selecting the virtual body that better matches the ideal and current body size and shape five times respectively. The order of body presentation is randomized across trials. In each trial, aesthetic features of the body - but the body parameters - are manipulated. | Participants are immersed in a virtual room in front of a door. The task requires adjusting the sliding door to determine the minimal aperture they need to pass through without rotating their shoulders. The task is proposed four times: two times starting with a closed door, and two times starting with a completely open door. |
| 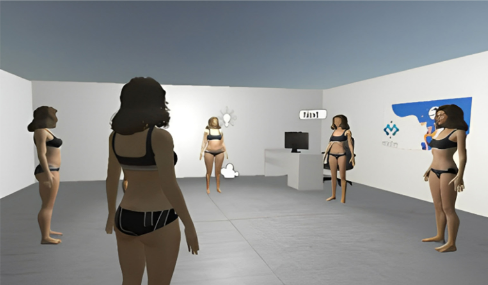 | 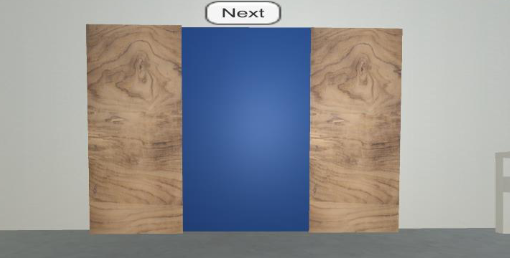 |

*Figure 2*. Scene from BIVRA System (Brizzi et al.,2024) and APE_VR (Brizzi & Romano, 2022).

*Embodiment Strength -* Body illusion strength was assessed after 90 seconds of synchronous multisensory stimulation (Serino et al., 2019) at the beginning of the protocol. We used visuo-tactile synchronous stimulation where the patient sees a virtual ball touching the virtual body’s abdominal area while the experimenter applies the same stimulation on her physical body with the controller. Embodiment was assessed using a visual analog scale (VAS) from 0 to 100 proposed directly inside the virtual environment (Porras-Garcia et al., 2020). The question was *Indicate on a scale from 0 (not at all) to 100 (totally) how much you agree with the following statement: ‘I perceived the virtual body as if it were my own*.

**Virtual reality setup**

The patient wore the VR headset (Oculus Quest 2) and had to handle one of the controllers while the other controller was managed by the experimenter. The motion tracking system was developed using Azure Microsoft Kinect to synchronize the patient's and the artificial body's movements (https://azure.microsoft.com/it-it/products/kinect-dk).

The virtual body was created using Make Human software (https://www.makehumancommunity.org) and was designed based on prior studies to represent a normal-weight body (*Figure 3*; Di Lernia et al., 2023; Keizer et al., 2016; Serino et al., 2017). The virtual scenario was developed in Unity 3D (https://unity.com). The virtual environment was a simple room with a large mirror on the front wall and no furniture of any kind. The mirror was large enough to reflect each limb of the body without showing the face and was placed approximately 1.5m in front of the user's position (Porras-Garcia et al., 2020).

|  | Virtual Body |  |
| --- | --- | --- |
| *Frontal view* | *Lateral view* | *Back view* |
| 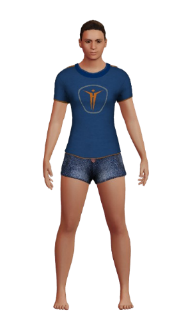 | 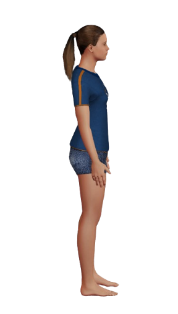 | 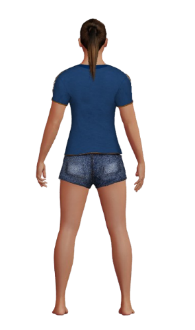 |

**Figure 3**. Virtual body measures. Waist-to-height ratio equal to 0.4606; Front chest distance = 26.58 cm; Bust circumference = 82.99 cm; Underbust circumference = 66.57 cm; Waist circumference = 73.95 cm; Nape to waist = 38.34 cm; Waist to hips = 19.01 cm; Shoulder distance = 14.14 cm; Age = 25, Muscle = 50%, Height = 160.54 cm; Hips = 96.72. These parameters were based on previous research (Di Lernia et al., 2023; Keizer et al., 2016; Serino, et al., 2017).

**** Functionality-Focused Mirror Exposure Procedure (FME) – Script**

Now I invite you to stand in front of this mirror. I will ask you to look carefully at different parts of your body, starting from the upper parts and then gradually working your way down. I'm going to ask you to focus on what each part of your body has allowed you to do in the past, and what it allows you to experience every day. In this exercise, you will be accompanied by my voice.

Your experience today may be different from the times you've looked in the mirror at home or elsewhere. In the past, exposure to the mirror may have been brief. In addition, you may have focused primarily on your "imperfections," i.e., those parts of your body that you don't like, fostering self-critical thoughts while looking at your reflection in the mirror. Because of our typical and habitual ways of examining ourselves when we are in front of the mirror, it is likely that we will experience critical emotions and thoughts, even during the exercise we will do together. That's okay, that's completely acceptable. While it can be unpleasant, research suggests that this discomfort is short-lived and that mirror exposure can be beneficial over time, even for those who experience discomfort in the process.

The purpose of this procedure is not to avoid or try to suppress your emotions. In case emotions and thoughts arise that make you feel uncomfortable, try to notice them, and then try to redirect your attention to my voice and instructions: think of all the positive things that your body has allowed you and allows you to do and experience.

The goal of this exercise is to help you recognize and appreciate what your body does for you in your daily life.

Start by making sure you see your entire body in the mirror.

Now start looking downwards and stopping to look at your chest. Think not only about your outward appearance but about what's under your skin. [Pause]

Think about the vital organs that are housed in your chest cavity. [Pause]

Can you hear your heartbeat? [Pause] Do you feel your lungs expand and contract with every breath you take? [Pause] Think of the ribs, which help protect those vital organs. [Pause] Think about how your heart rate and breathing speed help you handle physical challenges, giving your muscles more oxygen when needed, and how they slow down to allow you to be calm when you're at rest. [Pause]

What other internal organs that help you survive are protected by your chest? [Pause]

Now, move your eyes a little further downwards, at the level of your stomach. [Pause] How are you feeling? [Pause] Take some time to think about the function of your stomach. [Pause] What does it allow you to do regularly? [Pause] How does it keep you healthy? [pause] How do you feel when you respect the messages he sends you? [Pause] How do you feel when you ignore what your stomach is telling you? [Pause]

Think about how your stomach helps break down basic nutrients, so you can have the vitamins and minerals your body needs, how it helps you break down the amino acids that form proteins that are essential for muscles and other organs in the body, how it allows you to break down fats for hormonal function to ensure brain function, and how it helps you break down the glucose that provides the body and brain with fundamental and necessary energy. [Pause]

Now look at your shoulders and arms. [Pause] What do these body parts help in your daily life? [Pause] Do you have hobbies or activities such as a sport you like to do where you rely on your arms and shoulders? [Pause]

Now shift your attention to your lower arms, wrists, hands, all the way to your fingertips. [Pause] What have these parts, designed for dexterity, fine movements, and touch, allowed you to do and still do? [Pause] How many items have you picked up and put down? [Pause] How have these body parts helped you in the past to carry out your daily activities? [Pause] Have you typed, written, or texted someone you cared about? [Pause]

Did they allow you to play an instrument or play a game? [Pause] Or draw or paint? [Pause] Did you build anything? [Pause] Have you opened jars and containers for soap, and toothpaste, which have helped you take care of your body? [Pause]

Now, think about the pleasant feeling you had when you held something you held in your arms, hands, and fingertips. What has allowed you to appreciate your sense of touch? [Pause] Try to imagine the sensation of running your hand over a soft, fluffy surface, whether it's your pet's, a loved one's hair, or a beloved object. [Pause]

Now think about all your arms: from your shoulder to your fingertips [Pause] How do they work together? [Pause] Do they work together in perfect harmony? [Pause] When you move your arms and hands, do you see how the parts move and work together? [Pause] What things did you enjoy doing that your arms, wrists, hands, and fingers allow you to do every day? [Pause].

Try to remember a positive time when you held a friend, family member, or pet, or caressed a loved one. [Pause].

Finally, direct your gaze towards your legs. We often criticise ourselves for our upper legs and thighs, rather than focusing on their strength and capacity. [pause]. Think about how many good things have enabled and enabled you to have strong legs [Pause]. What activities have helped and helped you do? [Pause]. For example, did they allow you to go for walks in nature, ride a bike, run, or ski? [Pause]. Did they allow you to take a dance class or just have fun dancing with your friends? [Pause]. Did they help you sit up and get up today? Did they help you to go up and down the stairs? [Pause]. Now think about how your legs work together, from your thighs to your knees, calves, ankles, and feet. [Pause]

Think about every bone, joint, ligament and tendon that makes up your leg. [Pause] What do these body parts working together allow you to do? [Pause] Do they all serve a purpose together? [Pause] What do your legs allow you to do? [Pause] How do your legs support you? [Pause] Did your legs and feet carry you from one place to another? [Pause] To what safe and positive place did your legs and feet take you? [Pause]. Where did your legs and feet take you today? in the past? [Pause]. Where do you want those legs and feet to take you in your future? Try to imagine what your legs will allow you to do in the future, where they can take you and what life goals they will help you achieve [Pause].

Now, it is time to look at your whole body, to observe and appreciate all its parts, together [Pause].

We often spend time criticising our own bodies and judging the bodies of others for their appearance. We are socialized to this by others, such as family, friends and the media. However, this prevents us from taking the time to appreciate our body for what it can do for us, for the experiences it gives. Examining your body as a whole, think about the things you are grateful for that it has allowed you and will allow you to do and experience. [Pause]

Think back to a time when you felt proud of your body's capabilities or strength. What were you doing at that moment? [Pause] What are you grateful to your body for today? [Pause] What will you be able to do tomorrow? [pause] What about next week? next year? [Pause] Focus on a moment when you showed kindness to your body. [Pause] How did it make you feel to take care of yourself? [Pause] Now, try to imagine a future version of yourself: what goals can your body allow you to achieve? [Pause] What characteristics or habits do you think this future self that achieves all these goals might have?

**Qualitative data – Briefing and debriefing**

**Pre-Assessment**

- **What do you expect from this experience?**
- **Do you think using virtual reality will help improve your relationship with your body?**
- **Have you ever used virtual reality before?**

**Post - Final session**

- **How did it go?**
- **How did you feel?**
- **Do you have any suggestions?**
- **Do you think virtual reality will help improve your relationship with your body?**
- **Would you participate in another protocol like this?**

**Table 1.** Multidisciplinary therapeutic- rehabilitation program (Brusa et al., 2023).

| Standard therapeutic- rehabilitation program | |
| --- | --- |
| *Activity* | *Goal* |
| Individual dietary programme | Personalized diet aimed at body weight restoration and monitoring of eating behaviors. |
| Nutritional educational | Psychoeducation sessions about the importance of eating, food consumption, and calories. |
| Individual psychological sessions | Sessions aim to make the patient aware of her condition, recognizing the aspects that contributed to the onset of symptoms and understanding the necessary steps to facilitate change. |
| Physical activity | Walks according to the DCA ergometer and postural gymnastics once a week. |
| Educational groups | Psychoeducational activity, motivational group, therapeutic group, reading group. |
| Psychiatric evaluations | Weekly psychiatric evaluation during the multidisciplinary visit. |
| Pharmacological therapy | Citalopram 40 mg and Olanzapine 2.5 mg |

**References**

1. Alleva, J. M., Martijn, C., Van Breukelen, G. J., Jansen, A., & Karos, K. (2015). Expand Your Horizon: A programme that improves body image and reduces self-objectification by training women to focus on body functionality. *Body Image*, *15*, 81–89. <https://doi.org/10.1016/j.bodyim.2015.07.001>
2. Brizzi, G., & Romano, D. (2022) Dealing with your body (representation):a novel virtual reality-based assessment tool [Doctoral dissertation]. *ResearchGate*. DOI:[10.13140/RG.2.2.22587.69926](http://dx.doi.org/10.13140/RG.2.2.22587.69926)
3. Brizzi, G., Riva, G., & Romano, D. (2024). The Body Image Virtual Reality Assessment (BIVRA): Measuring the body representation through virtual reality. *Journal of Neuropsychology*. <https://doi.org/10.1111/jnp.12366>
4. Brooks, J., Walker, D. C., & Murray, K. (2023). What can my body do for me? Guided body-functionality mirror gazing task improved college women’s body appreciation and body functionality orientation. *Journal of American College Health*, 1–10. <https://doi.org/10.1080/07448481.2023.2209195>
5. Brusa, F., Scarpina, F., Bastoni, I. *et al.* Short-term effects of a multidisciplinary inpatient intensive rehabilitation treatment on body image in anorexia nervosa. *J Eat Disord* **11**, 178 (2023). <https://doi.org/10.1186/s40337-023-00906-9>
6. Cinelli G, Criscuolo M, Bifone C, Chianello I, Castiglioni MC, De Lorenzo A, Di Renzo L, Tozzi AE, Vicari S, Zanna V. Food Addiction in a Group of Italian Adolescents Diagnosed for Eating Disorder. Nutrients. 2020; 12(5):1524. <https://doi.org/10.3390/nu12051524>
7. Clausen, L., Rosenvinge, J. H., Friborg, O., & Rokkedal, K. (2011). Validating the Eating Disorder Inventory-3 (EDI-3): A Comparison Between 561 Female Eating Disorders Patients and 878 Females from the General Population. Journal of psychopathology and behavioral assessment, 33(1), 101–110. <https://doi.org/10.1007/s10862-010-9207-4>
8. Cuzzolaro, M., Vetrone, G., Marano, G., & Garfinkel, P. (2006). The Body Uneasiness Test (BUT): Development and validation of a new body image assessment scale. *Eating and Weight Disorders - Studies on Anorexia Bulimia and Obesity*, *11*(1), 1–13. <https://doi.org/10.1007/bf03327738>
9. Dakanalis, A., Timko, A. C., Clerici, M., Riva, G., & Carrà, G. (2017). Objectified Body Consciousness (OBC) in Eating Psychopathology: Construct Validity, Reliability, and Measurement Invariance of the 24-Item OBC Scale in Clinical and Nonclinical Adolescent Samples. *Assessment*, *24*(2), 252-274. <https://doi.org/10.1177/1073191115602553>
10. Gardner, R. M., Jappe, L. M., & Gardner, L. (2008). Development and validation of a new figural drawing scale for body‐image assessment: the BIAS‐BD. *Journal of Clinical Psychology*, *65*(1), 113–122. https://doi.org/10.1002/jclp.20526
11. Gardner, R. M., & Brown, D. L. (2014). Body size estimation in anorexia nervosa: a brief review of findings from 2003 through 2013. *Psychiatry research*, 219(3), 407-410. <https://doi.org/10.1016/j.psychres.2014.06.029>
12. Griffen, T. C., Naumann, E., & Hildebrandt, T. (2018). Mirror exposure therapy for body image disturbances and eating disorders: A review. *Clinical Psychology Review*, *65*, 163-174. <https://doi.org/10.1016/j.cpr.2018.08.006>
13. Hildebrandt, T., Loeb, K., Troupe, S., & Delinsky, S. (2012). Adjunctive mirror exposure for eating disorders: A randomized controlled pilot study. *Behaviour Research and Therapy*, *50*(12), 797–804. https://doi.org/10.1016/j.brat.2012.09.004
14. Irvine, K. R., McCarty, K., McKenzie, K. J., Pollet, T. V., Cornelissen, K. K., Tovée, M. J., & Cornelissen, P. L. (2018). Distorted body image influences body schema in individuals with negative bodily attitudes. *Neuropsychologia*, *122*, 38–50. <https://doi.org/10.1016/j.neuropsychologia.2018.11.015>
15. Keizer, A., Van Elburg, A., Helms, R., & Dijkerman, H. C. (2016). A virtual reality full body illusion improves body image disturbance in anorexia nervosa. *PLoS ONE*, *11*(10), e0163921. <https://doi.org/10.1371/journal.pone.0163921>
16. Kerr‐Gaffney, J., Harrison, A., & Tchanturia, K. (2019). Eye‐tracking research in eating disorders: A systematic review. *International Journal of Eating Disorders*, 52(1), 3-27. <https://doi.org/10.1002/eat.22998>
17. Key, A., George, C. L., Beattie, D., Stammers, K., Lacey, H., & Waller, G. (2002). Body image treatment within an inpatient program for anorexia nervosa: The role of mirror exposure in the desensitization process. *International Journal of Eating Disorders*, *31*(2), 185–190. <https://doi.org/10.1002/eat.10027>
18. Klimek, P., Wei, B., & Blashill, A. J. (2020). Exploring moderators of mirror exposure on pre- to post changes in body image outcomes: Systematic review and meta-analysis. *Eating Disorders*, *30*(1), 77–98. <https://doi.org/10.1080/10640266.2020.1791665>
19. Lernia, D. D., Serino, S., Tuena, C., Cacciatore, C., Polli, N., & Riva, G. (2023). Mental health meets computational neuroscience: A predictive Bayesian account of the relationship between interoception and multisensory bodily illusions in anorexia nervosa. International journal of clinical and health psychology : IJCHP, 23(4), 100383. <https://doi.org/10.1016/j.ijchp.2023.100383>
20. Luethcke, C. A., McDaniel, L., & Becker, C. B. (2011). A comparison of mindfulness, nonjudgmental, and cognitive dissonance-based approaches to mirror exposure. *Body Image*, *8*(3), 251–258. <https://doi.org/10.1016/j.bodyim.2011.03.006>
21. Malighetti, C., Chirico, A., Serino, S., Cavedoni, S., Matamala-Gomez, M., Stramba-Badiale, C., Mancuso, V., Corno, G., Polli, N., Cacciatore, C., Persani, L., & Riva, G. (2020). Manipulating body size distortions and negative body-related memories in patients with Anorexia Nervosa: A virtual reality-based pilot study. *Annual Review of CyberTherapy and Telemedicine, 18, 177-181*.
22. McKinley, N. M., & Hyde, J. S. (1996). The objectified body consciousness scale: Development and validation. *Psychology of Women Quarterly, 20*(2), 181–215. [https://doi.org/10.1111/j.1471-6402.1996.tb00467.x](https://psycnet.apa.org/doi/10.1111/j.1471-6402.1996.tb00467.x)
23. Petkova, V. I., & Ehrsson, H. H. (2008). If I Were You: Perceptual illusion of body swapping. *PLoS ONE*, *3*(12), e3832. <https://doi.org/10.1371/journal.pone.0003832>
24. Porras-Garcia, B., Ferrer-Garcia, M., Serrano-Troncoso, E., Carulla-Roig, M., Soto-Usera, P., Miquel-Nabau, H., Olivares, L. F. C., Marnet-Fiol, R., De La Montaña Santos-Carrasco, I., Borszewski, B., Díaz-Marsá, M., Sánchez-Díaz, I., Fernández-Aranda, F., & Gutiérrez-Maldonado, J. (2021). AN-VR-BE. A Randomized Controlled Trial for Reducing Fear of Gaining Weight and Other Eating Disorder Symptoms in Anorexia Nervosa through Virtual Reality-Based Body Exposure. *Journal of Clinical Medicine*, *10*(4), 682. <https://doi.org/10.3390/jcm10040682>
25. Serino, S., Pedroli, E., Keizer, A., Triberti, S., Dakanalis, A., Pallavicini, F., Chirico, A., & Riva, G. (2015). Virtual Reality body swapping: a tool for modifying the allocentric memory of the body. *Cyberpsychology Behavior and Social Networking*, *19*(2), 127–133. <https://doi.org/10.1089/cyber.2015.022>
26. Serino, S., Chirico, A., Pedroli, E., Polli, N., Cacciatore, C., & Riva, G. (2017). Two-phases innovative treatment for anorexia nervosa: the potential of virtual reality body-swap. *Annual Review of CyberTherapy and Telemedicine* *15*, 111-115.
27. Serino, S., Polli, N., & Riva, G. (2018). From avatars to body swapping: The use of virtual reality for assessing and treating body‐size distortion in individuals with anorexia. *Journal of Clinical Psychology*, *75*(2), 313–322. <https://doi.org/10.1002/jclp.22724>
28. Stice, E., & Presnell, K. (2007). *The Body Project: Promoting Body Acceptance and Preventing Eating Disorders Facilitator Guide*. <https://psycnet.apa.org/record/2007-10085-000>
29. Thompson, J. K. (1999). Physical Appearance State and Trait Anxiety Scale. *APA, Washington, DC*.
30. Tylka, T. L., & Wood-Barcalow, N. L. (2015). The Body Appreciation Scale-2: item refinement and psychometric evaluation. *Body image*, *12*, 53-67. <https://doi.org/10.1016/j.bodyim.2014.09.006>
31. Walker, D. C., & Murray, K. (2022). A pilot clinical case series of Functionality-Focused mirror exposure in women with clinically elevated body dissatisfaction. *Cognitive and Behavioral Practice*, *31*(1), 90–108. <https://doi.org/10.1016/j.cbpra.2022.05.001>
